# Barriers to sexual and reproductive healthcare services as experienced by female sex workers and service providers in Dhaka city, Bangladesh

**DOI:** 10.1371/journal.pone.0182249

**Published:** 2017-07-31

**Authors:** Tasnuva Wahed, Anadil Alam, Salima Sultana, Monjur Rahman, Nazmul Alam, Monika Martens, Ratana Somrongthong

**Affiliations:** 1 College of Public Health Sciences, Chulalongkorn University, Bangkok, Thailand; 2 Research to Policy Limited, Mirpur, Dhaka, Bangladesh; 3 Health Systems and Population Studies Division, icddr,b, Dhaka, Bangladesh; 4 HIV/AIDS Sector, Save the Children, Gulshan, Dhaka, Bangladesh; 5 University of Montreal Hospital Research Center (CRCHUM), Montreal, Quebec, Canada; 6 Faculty of Health, Medicine and Life Sciences, Maastricht University, Maastricht, Netherlands; Public Library of Science, FRANCE

## Abstract

**Objectives:**

This study aimed to identify the barriers female sex workers (FSWs) in Bangladesh face with regard to accessing sexual and reproductive health (SRH) care, and assess the satisfaction with the healthcare received.

**Methods:**

Data were collected from coverage areas of four community-based drop-in-centers (DICs) in Dhaka where sexually transmitted infection (STI) and human immunovirus (HIV) prevention interventions have been implemented for FSWs. A total of 731 FSWs aged 15–49 years were surveyed. In addition, in-depth interviews (IDIs) were conducted with 14 FSWs and 9 service providers. Respondent satisfaction was measured based on recorded scores on dignity, privacy, autonomy, confidentiality, prompt attention, access to social support networks during care, basic amenities, and choice of institution/care provider.

**Results:**

Of 731 FSWs, 353 (51%) reported facing barriers when seeking sexual and reproductive healthcare. Financial problems (72%), shame about receiving care (52.3%), unwillingness of service providers to provide care (39.9%), unfriendly behavior of the provider (24.4%), and distance to care (16.9%) were mentioned as barriers. Only one-third of the respondents reported an overall satisfaction score of more than fifty percent (a score of between 9 and16) with formal healthcare. Inadequacy or lack of SRH services and referral problems (e.g., financial charge at referral centers, unsustainable referral provision, or unknown location of referral) were reported by the qualitative FSWs as the major barriers to accessing and utilizing SRH care.

**Conclusions:**

These findings are useful for program implementers and policy makers to take the necessary steps to reduce or remove the barriers in the health system that are preventing FSWs from accessing SRH care, and ultimately meet the unmet healthcare needs of FSWs.

## Introduction

The number of female sex workers (FSWs) is underreported in global population data. Latin America and Sub Saharan Africa have a higher prevalence of FSWs than other regions of the world (between 0.2% and 7.4%, and 0.4% and 4.3%, respectively); in comparison, the Asian rate is between 0.2% and 2.6%, the ex-Russian Federation rate between 0.1% and 1.5%, Eastern European rate between 0.4% and 1.4%, and Western European rate between 0.1% and 1.4% [1]. In 2009, an estimated 74,300 FSWs (0.22% of the female population in Bangladesh aged 15–49) were operating in brothels, hotels and residential settings, and on the streets of Bangladesh [2].

Sexual and reproductive health (SRH) problems, such as unwanted pregnancies, frequent abortions, maternal health problems, and sexually transmitted infections (STIs) are prevalent among FSWs [3–8]. Female sex workers are a marginalized and highly stigmatized group in society due to poverty, low education levels, and social rejection [9]. In most countries of the world, sex work is either illegal or semi-legal and for this reason, FSWs are usually excluded from the formal healthcare system [10,11]. This isolation has repercussions on their health and wellbeing and SRH in particular [11]. This makes them particularly vulnerable when seeking care and services offering contraceptives, abortion, maternal health, and treatment for STIs. Currently, increasing attention has been given to the goal of universal health coverage (UHC) and the important principle in healthcare ethics of ‘equal access to health care on the basis of equal need, free at the point of delivery’ [12]. Thus, to achieve UHC and to improve the health and well-being of vulnerable and marginalized populations, FSWs need special attention so that they can acquire access to the necessary health services.

A study conducted in Africa showed that 54 projects were focusing on supplying HIV prevention and STIs services to FSWs [13]. Similarly, health programs for FSWs in Bangladesh are largely focused on STI/HIV prevention, with very limited services available to address SRH treatment needs [14]. The most common barriers to service utilization by female sex workers in different parts of the world are long waiting hours, unknown or inconvenient location of clinics, lack of confidentiality and discrimination by healthcare providers, poor communication between service providers, stigma, shame, or fear of exposure to the public as a sex worker [15–17]. So far, only one reported Bangladeshi study on the use of HIV interventions among brothel-based FSWs has explained the barriers to service utilization from both a service recipient and a service delivery point of view. According to this study, the restrictions created by the ‘Sordarnis’ (a senior FSW who acts as the leader of a group of FSWs) were a major barrier to clinical service access by FSWs [18]. Supply-related barriers included lack of medicines and inadequate supply of condoms [18]. Furthermore, limited maternal and child health services, inadequate STIs service provision, and lack of a proper referral system were identified as the main barriers to providing satisfactory services to FSWs [18]. No prior studies in Bangladesh have investigated barriers to accessing SRH services experienced by hotel, residence, and street-based FSWs. Moreover, informal consultations with key STI/HIV program implementers targeting FSWs in Bangladesh have unveiled that barriers to health service provision for FSWs have not been well documented and reported.

Besides identifying barriers to accessing services, satisfaction upon utilizing these services also affects whether an individual will return, refer others, and comply with treatment. Poor satisfaction with services can be also be an important barrier to service utilization [19]. One of the goals of health systems is to assess patient satisfaction on non-medical aspects, such as dignity/respect, privacy, autonomy, confidentiality, prompt attention, access to social support networks during care, basic amenities, and choice of institution/care provider [20]. People’s non-medical aspects of care are contributing factor to achieve good performance of a health system.

The likelihood of having ineffective health programs in place increases when key policy decisions are not based on the successes and pitfalls of interventions of the past. Therefore, this study aims to determine the barriers to accessing SRH care from the view point of FSWs and service providers. Furthermore, this paper measures the healthcare satisfaction level of the FSWs who have sought SRH services, including access to contraceptives, access to abortion, STI treatments, and maternal health services. The findings are useful for developing recommendations to improve existing SRH related interventions by overcoming barriers as well as to undertake new activities or actions in near future.

## Materials and methods

### Study design

A mixed method study comprising quantitative and qualitative methods of data collection was carried out from July to December 2015.

### Study site

This study was conducted in Dhaka city in Bangladesh, where community-based drop-in-centers (DICs) have been established for STI/HIV prevention intervention. A DIC is an establishment designed to provide shelter to FSWs, as well as educational or counseling services, condom and lubricant distribution, and management of STIs. Approximately 25 DICs operate in Dhaka city, targeting around 6,032 street, hotel, and/or residence based FSWs through two consortia, the Bangladesh Women Health Coalition (BWHC) and the Durjoy Nari Songha (DNS), funded by Save the Children. We collected information on all DICs (n = 25) in Dhaka with the help of Save the Children Bangladesh. Three DICs were selected to administer a quantitative survey and one additional DIC was selected strategically (i.e. the DIC had the highest number of FSWs) for qualitative data collection. A total of four DICs participated. The reason for selecting an additional DIC to conduct qualitative IDIs with FSWs was to avoid interviewing the same FSWs twice, for both qualitative and quantitative purposes.

### Study population

The study population included street, hotel, and/or residence based FSWs of reproductive age (15–49 years). Service providers of the selected DIC were also included for qualitative IDIs.

### Sample size

#### Quantitative

We used a statistical formula, “n = z^2^pq/d^2^” [21] to calculate the sample size. Here, we considered z = 1.96 at 95% confidence interval, with p = 0.5 indicating when SRH related services utilization by FSWs was unknown, q = 1-p, and d = 0.05 (5% margin of error) and estimated that the required sample size (n) was 384. Considering a design effect of 1.5, the proposed sample size was 576. Considering a 20% non-availability and non-response rate, the required sample size was 720.

#### Qualitative

A total of 23 IDIs with 14 FSWs and 9 service providers were conducted. The interviews were conducted until saturation of themes was obtained.

### Sampling and recruitment

#### Quantitative

**Fig 1:**
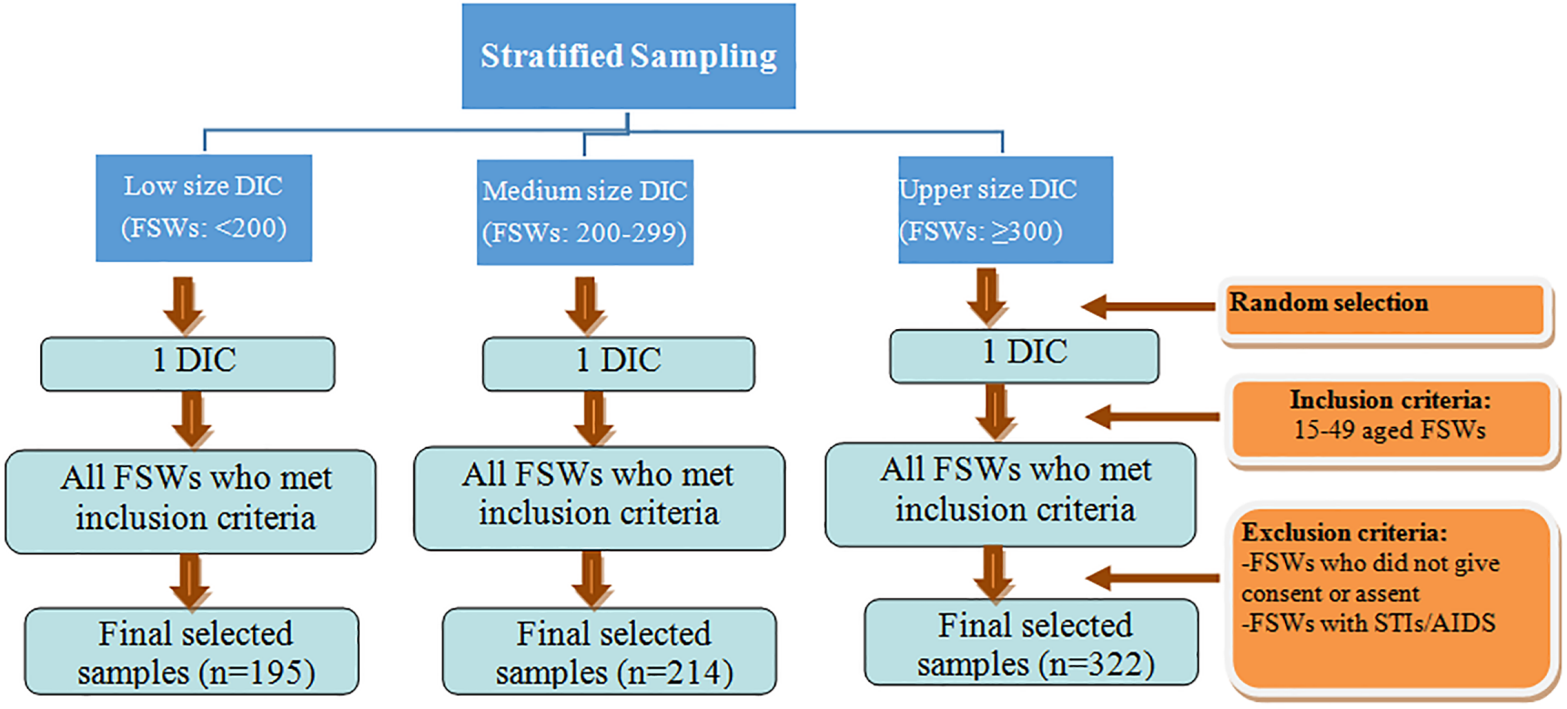
The stratified sampling technique.

The study sample was collected using a stratified sampling technique **(Fig 1)**. Administrative data from the DICs showed that the number of FSWs per DIC varied from 150–387. For representation of different population size of DIC, three separate lists were created for low, medium, and upper DICs. Drop-in centers with under 200 FSWs were considered low size; DICs with 200–299 FSWs were considered medium. The remaining DICs with ≥300 FSWs were considered upper DICs. One DIC per size group was randomly selected. Hence, a total of three DICs were included in the sample. All FSWs at the selected DICs who met the inclusion criteria were interviewed.

#### Qualitative

A purposive sampling technique was employed to select participants for IDIs. FSWs with prior experience of SRH care services were invited to participate in the IDIs. The DIC service providers identified FSWs who were either using or not using modern contraceptive methods (oral contraceptive pills, condom, injectables, intra-uterine devices, female sterilizations), or FSWs who had had an abortion, given birth to a child, or had been seen for an STI in the previous year. These FSWs were invited for an interview. Similarly, we conducted nine IDIs with purposively selected service providers of all four DICs, which included three paramedics, three DIC-coordinators, two supervisors, and one outreach worker **(Table 1)**. Respondents were selected according to availability and interest in participating in the interviews at a time convenient to them.

**Table 1:** Characteristics of qualitative in-depth interview participants.

Type of participants	Characteristics	Number of participants (f)
**a) Female sex workers (n = 14)**		
	**Age in years**	
	15–19	4
	20–24	4
	≥25	6
	**Completed years of schooling**	
	0–5	5
	≥6	9
	**Marital status**	
	Married	8
	Unmarried/divorced/separated/estranged husband	6
	**SRH experiences**	
	Currently using a modern contraceptive	10
	Had an abortion within the last year	8
	Had given birth in the last year	5
	Had an STI in the last year	4
**b) Service providers (n = 9)**		
	**Designation**	
	DIC paramedics	3
	DIC coordinators	3
	DIC supervisors	2
	Out-reach workers	1

DIC = drop-in-center

### Data collection tools

#### Quantitative questionnaire

A structured Bengali (local language) questionnaire was used to obtain information on whether respondents had utilized any SRH service in the last year, and on whether they had experienced barriers when utilizing SRH services. Questions related to satisfaction in the questionnaire were formed based on a review article which evaluated different survey tools on responsiveness to non-medical expectations and proposed the following elements: respect for dignity, autonomy, confidentiality, access to prompt attention, access to social support networks, basic amenities, and choice of institution/care providers [20]. Example questions from the questionnaire can be found in **S1 Table**. This questionnaire was field-tested by means of face to face interviews with 30 FSWs of a nearby DIC, which was not selected as a study site for the final interviews. The feedback generated in the field-testing was incorporated in the final questionnaire. On average, it took half an hour to complete a survey interview.

#### Qualitative guidelines

Two separate guidelines were prepared as given in **S1 File**. One guideline was used for IDIs with FSWs and another for interviews with service providers. A literature review was undertaken to form the interview guidelines. The guideline for FSWs contained a few domains, such as contraceptive use, abortion, maternal healthcare, STIs, and referral care. Issues concerning care-seeking practices and barriers faced when seeking SRH services were explored in those domains. Information was also obtained from service providers at the four DICs. More specifically, availability of SRH services, required logistics, barriers for FSWs in service delivery, and service provider experiences and suggestions were captured through IDIs with service providers. Similar to the quantitative questionnaires, these interview guidelines were pre-tested in a nearby DIC (excluded as a study site).

### Data collection procedure

#### Quantitative

A list of FSWs aged 15–49 years was prepared by the selected DICs. The outreach workers at the DICs were able to identify the FSWs, because they had regular contact with FSWs during condom distribution and health education sessions. Four data collectors, who were chosen because they had previous experience conducting health surveys, received additional orientation and training related to the data collection tool and this particular study population. The interviews were conducted in the Bengali language at the DIC and at the field level (parks, streets, and residential settings where FSWs are usually invited by pimps etc.). On average, each survey interview took half an hour.

#### Qualitative interviews

Most of the IDIs were conducted by the principal investigator herself as she had sufficient training and experience in qualitative research. However, one additional experienced qualitative interviewer was recruited to assist with the IDIs and to prepare the transcriptions of the interviews. The interviews were conducted at the DICs. An audio-recorder was used to record the interviews.

### Data analysis

#### Quantitative data

A web-based application was developed to capture data using ASP.Net and SQL server. These data were converted to SPSS format by means of the statistical software SPSS version 20 (SPSS Inc., Chicago, IL, USA) for data editing and analysis. Descriptive analyses were performed to understand socio-demographic characteristics of the FSWs, barriers to healthcare access, and SRH service related satisfaction from formal healthcare providers. Formal healthcare was defined in this study as ‘care from recommended healthcare sources, such as-public, private for profit, and private not for profit health facilities including healthcare by qualified providers from home’. A total of eight items were used to calculate satisfaction, namely dignity, privacy, autonomy, confidentiality, prompt attention, access to social support networks during care, basic amenities, and choice of institution/care provider [20]. A scoring system with a three-point satisfaction scale was generated in the following manner: highly satisfied/moderately satisfied = 2, satisfied = 1, less satisfied/not satisfied = 0. Based on these given scores, the minimum and maximum possible scores were 0 and 16, respectively. The proportion of respondents who scored >50% (i.e., respondents with a score between 9 and 16) was also calculated using descriptive statistics. Furthermore, one way ANOVA was used to measure the mean difference in satisfaction scores by different types of formal healthcare (e.g., public, for-profit private, not for profit private or not-for-profit private non-governmental organizations (NGOs), at home by skilled providers). The level of statistical significance was set at p<0.05.

#### Qualitative data

A descriptive content analysis procedure was applied for analysis of qualitative data [22–24]. At first, the transcripts were prepared in Bengali based on audio recording files. A code list was generated by the principal investigator through continuous reading of transcripts, revisiting transcripts to validate any confusion or inconsistencies, and revising the content. Two co-authors provided feedback to validate and finalize the codes. The qualitative data management software, ATLAS.ti, version 5.2 (Atlas.ti, GmbH, Berlin, Germany) was used for coding and compilation of data. Data were interpreted according to theme and sub-theme. Excerpts were used to express the voice of interview participants.

### Ethics approval

The Research Ethics Review Committee of Chulalongkorn University, Thailand,approved this study (Protocol No: 178.1/58). As most of the FSWs were illiterate, datacollectors read the whole consent paper to the participants. To avoid FSWs’ fear of identification and to maintain confidentiality, written consent was waived by the Research Ethics Review Committee of Chulalongkorn University. Therefore, verbal consent was collected from the adult FSWs before the start of the first interview. Informed consents from guardians following informed assents from the FSWs who were aged 15 to 17 years were obtained. Data collectors signed and dated the consent forms after obtaining verbal consent. Written consent was obtained from service providers before starting the IDIs.

## Results

### Status of survey interview

In the administrative database of the 3 selected DICs, 740 FSWs were recorded. Of them, 731 were interviewed and the remaining 9 FSWs were absent. All 731 respondents gave consent before participating in the interviews.

### Characteristics of respondents

#### Survey

Of the 731 FSWs interviewed, the mean age (±SD) was 23.53 (±7.05) years. More than half of them (53.5%) had been involved in the sex profession for at least five years. The mean (±SD) period of schooling was 2.7 (±3.08) years. The median monthly income was 10000 BDT (126.56 USD).

#### Qualitative interviews

The majority of the FSWs were under 25 years-of-age, had completed 6 or more years of schooling (n = 9), and were married (n = 8). Ten out of the 14 FSWs interviewed were using some type of modern contraceptive (oral contraceptive pills, condom, injectables, intra-uterine devices, female sterilizations). Respondents who had undergone an abortion (n = 8), given birth (n = 6), or experienced an STI (n = 4) in the last year were included in the interviews **(Table 1)**.

### Barriers to SRH service utilization

Overall, 51% of the 731 FSWs reported facing barriers while seeking SRH care in formal healthcare. The majority (72%) mentioned the cost of care or lack of money for services and feeling ashamed about seeking SRH services (52%) as barriers. Other barriers included the unwillingness of service providers to provide care to FSWs (40%), unfriendly behavior of providers (17%), lack of 24-hour service availability or seeking services outside of normal business hours (12%), and fear of hatred from providers (5.6%) **(Table 2)**.

**Table 2:** Perceived barriers to sexual and reproductive health service access in formal health facilities among female sex workers over the past year.

Variables	%
Types of barriers	n = 353
Costly/lack of money to access services or obtain medication	72.1
Ashamed to seek SRH services	52.3
Unwillingness of service providers to provide services	39.9
The unfriendly behavior of providers	24.4
Distance to care	16.9
Lack of 24-hour service availability/seeking services outside normal business hours	12.1
Fear of hatred from providers	5.6
Unavailability of transport	1.9
Do not know where to obtain care	0.8

SRH = sexual and reproductive health

The FSWs who participated in the IDIs explained in more detail about the barriers they faced when they needed SRH services. The following paragraphs from the qualitative interviews reflect these barriers.

#### Financial barriers

Lack of money to utilize services is a very common barrier. The respondents complained that the providers would only accept cash in advance of treatment. One FSW shared her experience:

“*They did not talk without money*. *I was dying*, *I had bleeding (vaginal)*, *I was so restless*. *I was saying that I have money in my bank account*. *That day was Thursday (last week day) and bank get closed at afternoon*. *I need to give the women (health provider) 3000 taka*. *Then*, *I send another mate to bring money*.*”* [ID-107, 26 years of age, not educated, involved in sex trade for 6 years]

#### Unfriendly behavior of the providers

The FSWs indicated that health providers behaved badly when they knew they were treating sex workers. If providers came to know about their patient’s profession in the sex trade, they would sometimes demand more money than usual, according to the FSWs. Moreover, the FSWs mentioned that service providers often refused to provide care once they knew they were dealing with sex workers. The FSWs who sought contraceptive services from a pharmacy mentioned that not only would the shop keepers refuse to provide services, but they would also scold them, and call local boys or the police to catch them. One woman said:

*“Sometimes they do not provide treatment if they know that we are sex workers*. *Sometimes they have unprofessional behavior*, *say*, *why do you do such work*? *Leave it*, *become a good woman*. *It is not right*. *We feel bad in such circumstances*.*”* [ID-101, 18 years of age, completed 10 years of schooling, involved in sex trade for 6 years]

#### Discrimination to and ashamed by FSWs for seeking SRH services

During the IDIs, FSWs mentioned that they often observed other women who had come in for treatment. The FSWs felt that the providers did not behave as nicely to them, as they did to other women. A few FSWs said that the providers delivered services in a hateful manner. The FSWs also perceived that it was not possible to talk openly about their health problems to other healthcare providers who were unaware of their profession, even though they felt they could talk more openly to the DIC providers. This was one of the reasons why FSWs do not seek external health services very often. Unmarried FSWs also expressed that feeling shame was a reason to not discuss their SRH needs with a qualified doctor. A girl voiced this in the following way:

“I only know that there is a hospital name ‘x’ (name is avoided due to ethical considerations). This is a good hospital. But I can’t go there because I went there to accompany my niece for her treatment. Doctor now knows that I am unmarried. If I go there later and tell him/her that I am pregnant, will I not feel bad? Am I lose all my dignity?”

[ID-108, 17 years of age, completed 8 years of schooling, involved in sex trade for 2 ½ months]

#### Lack of knowledge

A younger FSW indicated that she did not have any knowledge about contraceptive methods, except for ‘*femicon*’ (an oral pill). She further disclosed that she did not know when to use that pill or whether she could stop taking the pills when she was not sexually active with clients. Another FSW said she needed to go to STI clinic *‘x’ (name is avoided due to ethical considerations)* for STI treatment, but she did not know where the place was.

#### Distance

A few FSWs mentioned that traffic congestion and long travel distances to referral centers are barriers to accessing services.

### Respondents’ satisfaction level with formal healthcare

**Table 3** displays the level of satisfaction of the respondents with formal healthcare. As shown in **Table 3A**, a total of 447 FSWs accessed contraceptive services, 97 FSWs accessed abortion services, 97 accessed maternal healthcare, and 253 FSWs accessed STI services from formal healthcare sources. **Table 3A** also shows that a few respondents (0.4% to 10.3%) could not respond to any of the eight satisfaction units and were excluded from the analysis. Finally, 371, 77, 84, and 221 respondents provided responses on contraceptives, abortion, maternal healthcare, and STI services use, respectively and were included in the analysis **(Table 3B).**

**Table 3:** Proportion of respondents satisfied with SRH related formal healthcare in the past year.

**3A. Satisfaction units**	% of respondents by type of SRH care															
	Contraceptive services n = 447				Abortion services n = 97				Maternal healthcare n = 97				STIs n = 253			
	HS	S	NLS	NCCR	HS	S	NLS	NCCR	HS	S	NLS	NCCR	HS	S	NLS	NCCR
Respect	29.4	55.7	14.2	0.7	20.6	48.5	30.9	0	36.1	39.2	24.7	0	29.2	54.9	14.6	1.2
Privacy	32.6	52.6	14.4	0.4	24.7	45.4	29.9	0	21.6	51.5	26.8	0	25.3	56.1	17.8	0.8
Autonomy	33.7	49.7	15.5	1.1	35.1	35.1	29.9	0	36.1	44.3	19.6	0	32.4	51.0	15.8	0.8
Confidentiality	29.9	49.9	18.2	2.0	33.0	35.1	32.0	0	30.9	40.2	27.8	1.0	32.0	52.6	14.6	0.8
Prompt attention	26.5	50.6	21.6	1.3	30.9	35.1	33.0	1.0	33.0	39.2	25.8	2.1	26.1	55.3	17.4	1.2
Social network support	25.6	47.4	20.0	7.0	35.1	36.1	22.7	6.2	34.0	35.1	27.8	3.1	28.5	50.6	17.4	3.6
Basic amenities	18.9	50.3	25.6	5.2	22.7	34.0	39.2	4.1	23.7	33.0	39.2	4.1	20.9	53.8	20.9	4.3
Choice of health center/ healthcare providers	13.9	51.2	27.0	7.9	22.7	25.8	41.2	10.3	18.6	38.1	35.1	8.2	19.4	51.0	23.3	6.3
**3B. Summary scores**	% of respondents whose score indicated a >50% rate of satisfaction by type of SRH care															
	Contraceptive services n = 371				Abortion services n = 77				Maternal healthcare n = 84				STIs n = 221			
Given score	HS = 2, S = 1, NLS = 0, NCCR = α				HS = 2, S = 1, NLS = 0, NCCR = α				HS = 2, S = 1, NLS = 0, NCCR = α				HS = 2, S = 1, NLS = 0, NCCR = α			
Respondents having >50% scores (Score = 9 to 16)	38.0				36.4				41.7				36.2			

HS = highly satisfied, S = satisfied, NLS = not satisfied or less satisfied, NCCR = no comments or could not remember

^α^excluded from score calculation

The detailed percentage distribution on different satisfaction units **(Table 3A)** and overall satisfaction scores **(Table 3B)** by type of SRH service are described below.

#### Contraceptive services

Less or more half (e.g., respect: 55.7%, privacy: 52.6%, autonomy: 49.7%) of the 447 respondents who sought contraceptive services from formal healthcare stated that they were satisfied with the services on all the different satisfaction units **(Table 3A)**. However, only 38% of respondents (n = 371) reported an overall satisfaction score that was higher than 50% **(Table 3B)**.

#### Abortion services

Satisfaction with abortion services was much lower than for other categories. For example, only 56.7% and 48.5% respondents were satisfied or highly satisfied with the basic amenities and choice of health center/healthcare providers for service respectively. About one-third of the respondents were less or not satisfied with privacy (29.9%), confidentiality (32.0%), and prompt attention (33.0%) issues **(Table 2A)**. Only 36.4% of the FSWs reported satisfaction scores of between 9 and 16 for abortion services **(Table 3B)**.

#### Maternal healthcare

Except for privacy (21.6%), basic amenities (23.7%), and choice of health center/healthcare providers (18.6%), about one-third of the FSWs were highly satisfied on different units of satisfaction, such as respect (36.1%), autonomy (36.1%), confidentiality (30.9%), prompt attention (33%), and social network support (34.0%) **(Table 2A)**. Overall, 41.7% of 84 cases showed satisfaction scores of more than 50% **(Table 2B)**.

#### STI services

Although the percentages of less satisfied or not satisfied on the different satisfaction units were relatively low for STI services compared to abortion and maternal healthcare, overall, only 36.2% (n = 221) showed satisfaction scores of over 50% **(Table 2B)**.

### Differences in level of satisfaction by type and place of service

**Table 4** and **Table 5** describe the results of a one-way ANOVA. Data showed that there were significant mean differences on satisfaction scores across the formal healthcare services.

**Table 4:** Satisfaction scores with formal healthcare, by type of service.

Type of services	Sources of formal healthcare	n	Mean [95% Confidence Interval for Mean]	Std. Deviation	Std. Error	Minimum	Maximum
	Public hospitals	20	7.1000 [4.9531–9.2469]	4.58717	1.02572	.00	14.00
Contraceptive services	For profit private clinic/chambers	14	9.9286 [7.7627–12.0944]	3.75119	1.00255	2.00	16.00
	NGO clinic	264	8.2386 [7.8610–8.6163]	3.11623	.19179	.00	16.00
	Home by skilled provider	73	11.8904 [11.1239–12.6569]	3.28533	.38452	.00	16.00
	Total	371	8.9596 [8.5934–9.3258]	3.58691	.18622	.00	16.00
	Public hospitals	23	5.3478 [3.1405–7.5552]	5.10444	1.06435	.00	15.00
Abortion services	For profit private clinic/chambers	22	8.4545 [7.1158–9.7933]	3.01942	.64374	3.00	16.00
	NGO clinic	23	9.2174 [7.1478–11.2870]	4.78593	.99794	.00	16.00
	Home by skilled provider	9	10.7778 [7.9593–13.5962]	3.66667	1.22222	5.00	16.00
	Total	77	8.0260 [6.9697–9.0822]	4.65373	.53034	.00	16.00
	Public hospitals	21	6.4286 [4.1274–8.7298]	5.05541	1.10318	.00	15.00
MCH services	For profit private clinic/chambers	18	8.3333 [6.7515–9.9151]	3.18082	.74973	.00	16.00
	NGO clinic	42	9.1429 [7.9437–10.3420]	3.84816	.59378	.00	16.00
	Home by skilled provider	3	10.6667 [2.6813–18.6521]	3.21455	1.85592	7.00	13.00
	Total	84	8.3452 [7.4442–9.2463]	4.15221	.45304	.00	16.00
	Public hospitals	18	3.9444 [2.2778–5.6111]	3.35142	.78994	.00	14.00
STI services	For profit private clinic/chambers	12	8.4167 [6.1588–10.6746]	3.55370	1.02586	2.00	14.00
	NGO clinic	189	9.4762 [9.0235–9.9289]	3.15482	.22948	.00	16.00
	Home by skilled provider	2	13.5000 [-18.2655–45.2655]	3.53553	2.50000	11.00	16.00
	Total	221	9.0045 [8.5349–9.4742]	3.54260	.23830	.00	16.00

NGO = not-for-profit private, MCH = maternal and child health, STI = sexually transmitted infection

**Table 5:** Mean within group differences in satisfaction scores for formal healthcare.

Dependent Variable	Sources of formal healthcare	Sources of formal healthcare	Mean Difference [95% Confidence Interval]	Std. Error	Sig.
Satisfaction score on contraceptive services (n = 371)	Public hospitals (n = 20)	For profit private clinic/chambers	-2.82857 [-6.0245-.3674]	1.13796	.105
		NGO clinic	-1.13864 [-3.2657-.9884]	.75737	.521
		Home by skilled provider	-4.79041^*^ [-7.1052–-2.4756]	.82420	.000
	For profit private clinic/chambers (n = 14)	Public hospitals	2.82857 [-.3674–6.0245]	1.13796	.105
		NGO clinic	1.68994 [-.8254–4.2053]	.89562	.315
		Home by skilled provider	-1.96184 [-4.6378-.7141]	.95280	.239
	NGO clinic (n = 264)	Public hospitals	1.13864 [-.9884–3.2657]	.75737	.521
		For profit private clinic/chambers	-1.68994 [-4.2053-.8254]	.89562	.315
		Home by skilled provider	-3.65177^*^ [-4.8646–-2.4390]	.43184	.000
	Home by skilled provider (n = 73)	Public hospitals	4.79041^*^ [2.4756–7.1052]	.82420	.000
		For profit private clinic/chambers	1.96184 [-.7141–4.6378]	.95280	.239
		NGO clinic	3.65177^*^ [2.4390–4.8646]	.43184	.000
Satisfaction score on abortion services (n = 77)	Public hospitals (n = 23)	For profit private clinic/chambers	-3.10672 [-6.8122-.5988]	1.29479	.134
		NGO clinic	-3.86957^*^ [-7.5336–-.2055]	1.28033	.034
		Home by skilled provider	-5.42995^*^ [-10.3154–-.5445]	1.70710	.023
	For profit private clinic/chambers (n = 22)	Public hospitals	3.10672 [-.5988–6.8122]	1.29479	.134
		NGO clinic	-.76285 [-4.4683–2.9426]	1.29479	.951
		Home by skilled provider	-2.32323 [-7.2398–2.5933]	1.71798	.611
	NGO clinic (n = 23)	Public hospitals	3.86957^*^ [.2055–7.5336]	1.28033	.034
		For profit private clinic/chambers	.76285 [-2.9426–4.4683]	1.29479	.951
		Home by skilled provider	-1.56039 [-6.4458–3.3250]	1.70710	.841
	Home (n = 9)	Public hospitals	5.42995^*^ [.5445–10.3154]	1.70710	.023
		For profit private clinic/chambers	2.32323 [-2.5933–7.2398]	1.71798	.611
		NGO clinic	1.56039 [-3.3250–6.4458]	1.70710	.841
Satisfaction score on maternal healthcare services (n = 84)	Public hospitals (n = 21)	For profit private clinic/chambers	-1.90476 [-5.6183–1.8087]	1.30027	.546
		NGO clinic	-2.71429 [-5.8041-.3755]	1.08189	.107
		Home by skilled provider	-4.23810 [-11.3737–2.8975]	2.49853	.416
	For profit private clinic/chambers (n = 18)	Public hospitals	1.90476 [-1.8087–5.6183]	1.30027	.546
		NGO clinic	-.80952 [-4.0665–2.4474]	1.14042	.918
		Home by skilled provider	-2.33333 [-9.5429–4.8762]	2.52442	.836
	NGO clinic (n = 42)	Public hospitals	2.71429 [-.3755–5.8041]	1.08189	.107
		For profit private clinic/chambers	.80952 [-2.4474–4.0665]	1.14042	.918
		Home by skilled provider	-1.52381 [-8.4328–5.3852]	2.41919	.941
	Home (n = 3)	Public hospitals	4.23810 [-2.8975–11.3737]	2.49853	.416
		For profit private clinic/chambers	2.33333 [-4.8762–9.5429]	2.52442	.836
		NGO clinic	1.52381 [-5.3852–8.4328]	2.41919	.941
Satisfaction score on STI services (n = 221)	Public hospitals (n = 18)	For profit private clinic/chambers	-4.47222^*^ [-7.8259–-1.1186]	1.19027	.003
		NGO clinic	-5.53175^*^ [-7.7515–-3.3120]	.78782	.000
		Home by skilled provider	-9.55556^*^ [-16.2628–-2.8483]	2.38053	.001
	For profit private clinic/chambers (n = 12)	Public hospitals	4.47222^*^ [1.1186–7.8259]	1.19027	.003
		NGO clinic	-1.05952 [-3.7384–1.6194]	.95079	.743
		Home by skilled provider	-5.08333 [-11.9562–1.7896]	2.43932	.230
	NGO clinic (n = 189)	Public hospitals	5.53175^*^ [3.3120–7.7515]	.78782	.000
		For profit private clinic/chambers	1.05952 [-1.6194–3.7384]	.95079	.743
		Home by skilled provider	-4.02381 [-10.4205–2.3729]	2.27029	.373
	Home by skilled provider (n = 2)	Public hospitals	9.55556^*^ [2.8483–16.2628]	2.38053	.001
		For profit private clinic/chambers	5.08333 [-1.7896–11.9562]	2.43932	.230
		NGO clinic	4.02381 [-2.3729–10.4205]	2.27029	.373

**Table 4** shows that FSWs did not give the highest maximum score of 16 to any of the SRH services (contraceptive:14, abortion:15, maternal healthcare: 15, STI:14) in public hospitals. Overall, the mean plus SD for satisfaction scores for the different SRH services, contraceptives, abortion, MCH, and STIs were 8.95 ± 3.5, 8.02 ± 4.6, 8.34 ± 4.1, and 9.00 ± 3.5 respectively.

**Table 5** shows that women who received services in their home or the provider’s home had a significantly higher level of satisfaction compared to women who visited formal healthcare facilities, with the exception of maternal healthcare (p<0.05). The contraceptive users who reported getting services from home by skilled providers had obtained some significant positive mean difference scores compared to other sources. Abortion services in public hospitals had comparatively lower mean satisfaction scores (-3.87) than NGO services. No significant mean difference was found between the different types of formal healthcare sources and maternal healthcare related satisfaction scores. Compared to STI services in public hospitals, all other formal healthcare sources (e.g., NGO or for-profit private clinics) had significantly higher satisfaction scores.

### Barriers to service delivery

#### Inadequacy of services

Most of the DIC service providers felt that they were providing inadequate services. They indicated that they mainly provide STI/HIV prevention services to FSWs in the DIC. The outreach workers distribute condoms to FSWs, identify STI patients, and send those patients to the DIC. The paramedic provides standard management for STIs. Most of them mentioned that they could not manage some cases as they did not have regular blood testing capabilities in the DIC. A team of testing researchers visits the DIC every three months to collect blood samples from 6/7 patients to identify types of STIs, but this is not adequate considering the needs of the patients at the DIC.

#### Logistic requirements

According to the DIC service providers, the necessary logistic and organizational support to provide STI services was available to them. They autoclaving machine, gully pot, cotton, cotton pot, forceps, gloves and medicine for management of STIs. However, a few of them noted that autoclaving of the medical equipment takes approximately half an hour in the morning, even though they only own one autoclave which can sterilize equipment for five patients at the same time. For this reason, they cannot provide treatment when more than five patients arrive, even when a patient is severely ill. The DIC providers also mentioned that they do not have access to ultrasonography at the DIC, although they advise some patients to have an ultrasonogram.

#### Stigma in the community

The outreach workers and supervisors mentioned facing problems at the field level, when they distribute condoms to the FSWs. One provider cited:

“*the community people make comments on us on the road as we work for them (FSWs)*. *Though we do awareness building activity at community*, *people think we help them at their illegal work*” [ID-205, Designation: DIC Supervisor, 16 years of schooling]

#### Lack of availability of other SRH services

Regarding the other SRH related services besides STIs, all providers mentioned the lack of contraceptive services, menstrual regulation (a procedure for establishing safe non-pregnancy condition within 8 to 10 weeks after a missed period) or abortion services, and pregnancy and delivery services in the DIC, despite the obvious need. They also mentioned that contraceptive services at the DIC would help to prevent frequent abortions.

“*The FSWs become frequently pregnant*. *There is no abortion service here*, *but they (FSWs) want*. *Or*, *they can eat (take) something to prevent pregnancies*, *there is no pill (oral contraceptive pill) supply here*, *they are wanting pills*.” [ID-202, Designation: DIC paramedic, 12 years of schooling with 18 months paramedical training]

#### Challenges with treatment referral

The paramedic in this study said that FSWs who think they may be pregnant, they (FSWs) are usually suggested to buy pregnancy test strips. The DICs have a referral system if a pregnant FSW is identified. They have links to non-profit private organizations (e.g., Marie Stopes International, Bangladesh Association for Prevention of Septic Abortion) that provide maternal and reproductive health services. However, barriers to access exist, including the high cost of treatment at the referral center.

“*I give free STI treatment here*, *but if I send FSWs to other clinic or hospital or private chamber*, *they will not give it free*. *For public hospital*, *FSWs need money for transportation and entry ticket*.*”* [ID-205, DIC Coordinator, 16 years of schooling]

*“If I say at the referral centers that it is my girl (FSW)*, *they will take 200 BDT for ultra-sonogram*. *They have to also collect a card where 30 BDT payment is compulsory*. *After that*, *the treatment they get*, *they have to buy medicine from outside or from them*.*”* [ID-203, DIC Coordinator, 10 years of schooling]

According to the providers, the DIC provides 135 BDT (79 BDT = 1 USD) for each referral case. This amount does not cover the cost of diagnostic tests or medication in the referral centers.

Some of the providers identified problems with the referral system. In this system, a mobile team visits a community place outside the DIC, once in a week for a half a day. This is where FSWs with SRH-related problems are expected to attend for a checkup by a medical doctor. This doctor gives the FSW a referral slip if she needs to go to a referral center. The DIC providers who participated in the IDIs said that visiting only once a week and checking only 5 or 6 patients is insufficient. This mobile team only accepts a referral if the pregnant woman is under 24 years-of-age. Many pregnant women are older, and do not meet these eligibility criteria. One in-depth interview participant commented on the unsustainable referral practices of the mobile team:

“*I heard that it will not go on*. *See*, *it is not coming for many days*, *my girls (FSWs) are returning for not seeing them*. *One of my FSW is near to her expected date of delivery but link up (the referral activity name) is not coming for 3/4 weeks*.” [ID-206, DIC outreach worker, 5 years of schooling].

In addition, the providers said if they had to send the cases directly to the nearby referral centers without a referral slip issued by the mobile team, the FSWs would not receive any services. In such cases, the providers must send the FSW to a place a great distance away for treatment. Furthermore, the DIC providers were obliged to send cases further away when the nearest referral centers lacked the health staff to deal with this referral service. According to the providers, many FSWs do not know the location of referral centers. One of the providers pointed out that FSWs are not as inclined to talk as openly about their health problems to referral center providers as they would with DIC staff members.

#### Attitude and behavior of healthcare providers

The DIC staff disclosed that the FSWs would not readily tell other healthcare providers about their profession. If they do disclose their profession, doctors do not have a good attitude towards them. Doctors usually ask many personal questions; for example, who is the father of this baby or why do they do this profession. Sometimes, the doctors start scolding them, and are unwilling to provide services or demand higher than routine fees.

*“While I accompanied with FSWs in health centers*, *I observed that the doctor behavior is different for the general women than FSWs*. *They gave the general women a good seat to sit*, *showing a good behavior*, *and make them understand the prescription nicely*.*”* [ID-206, DIC outreach worker, 5 years of schooling]

One provider at the DIC mentioned that she could not tell her family members that she is providing services to FSWs, because they would not accept it. Even the provider herself had difficulty coming to terms with her job at the beginning of her career.

#### Problems with law enforcement persons

As the police are aware that DIC service providers work with FSWs, they sometimes follow outreach workers when they go out into the field for condom distributions. These police officers later raid the places the outreach workers have been to, which creates a serious barrier to service delivery to the FSWs. Moreover, police are frequently conducting raids at different hotels. For this reason, services targeting hotel-based sex workers are being interrupted.

#### Lack of government support

One provider said that due to the low prevalence of HIV in Bangladesh, the government is paying less attention to HIV prevention programs in Bangladesh. As a consequence, international donors are reducing their funding for DIC activities.

#### Workload

The management of the DICs recently decided to close several DICs due to funding issues. A few providers mentioned that they had to increase the numbers of targeted FSWs after other DICs closed, making their own workload three times higher than usual. The reduction of staff was also reported in DICs where the duties of fired staff members were distributed among the outreach workers, and as a result, these staff members carry an extra burden of work. One participant in the IDIs mentioned that an outreach worker in her DIC started her working day at 2:00 pm, which is problematic as many FSWs are already in need of services in the morning.

#### Cultural barriers

A few providers mentioned that religious leaders of the mosque, neighbors, and other residences close to the DIC make objections from time to time about the services provided to FSWs in DICs.

## Discussion

This study highlighted that most the FSWs who sought formal healthcare for sexual or reproductive health services faced barriers and experienced moderate to low levels of satisfaction. Financial constraints and shame were the most common barriers identified by the FSWs themselves, whilst the unavailability of SRH services targeting FSWs and an inadequate referral system were identified as the main barriers by the service providers.

One of the goals of any health system is fair financial contribution to healthcare with the aim that individuals or households will not be burdened with catastrophic healthcare payments, and where the poor should be subsidized for their health-related costs by the rich [25]. Similar to our study, lack of money, the high cost of healthcare, and unaffordable fees for sex workers have been common problems reported in different parts of the world, such as in Nepal, China, Laos, Vietnam, and Russia [15,16,26–29]. Several studies have similarly indicated that FSWs felt shame about receiving services together with the general population, and that healthcare providers had a judgmental attitude toward them [17,26,30]. Stigma, discrimination, and fear of recognition was felt by FSWs, especially when healthcare providers asked them personal questions about the sex trade and their sexual history [16]. A study conducted in Laos in 2012 showed that FSWs did not even feel comfortable in the DICs that were established within public hospitals, as the chance of being seen by the general public was higher [15]. In Bangladesh, private DICs are available where the only patients are FSWs. However, comprehensive SRH services are not available in these DICs, only STI/HIV prevention services. A lack of availability of other SRH services (e.g. contraceptives, menstrual regulation/abortion, maternal healthcare) was identified as one of the key barriers to accessing SRH care for FSWs, and our findings are similar to the findings of another study conducted in Bangladesh in 2014 [14]. A referral system designed to overcome this barrier and to connect FSWs with the health system is a good option to improve access to care [29]. The DICs in Dhaka city have already developed a referral system with other NGOs. However, this referral system has proven to be weak due to inadequate referral support, the financial charge at referral centers, unsustainable referral provision, and referral center locations unknown to the FSWs.

Patient satisfaction is an important indicator for assessing the quality of a healthcare service [31]. The formal healthcare system in urban areas of Bangladesh requires the engagement of three types of participants: i) public clinic/hospitals, ii) NGOs (not-for-profit), and iii) private for profit clinic/hospitals. Formal health services also can be in homes by skilled medical professionals or community health workers. In our study, most of the respondents gave low satisfaction scores for formal healthcare. Several studies identified the need to maintain privacy and confidentiality in order to facilitate the acceptance of SRH services or increase the utilization of healthcare clinics [16,32–34].

In our study, although the majority of the respondents were just ‘satisfied’ (i.e. neither highly satisfied, less satisfied, or not satisfied) with privacy and confidentiality in the healthcare system, a good proportion of women were less or not satisfied with privacy and confidentiality in formal healthcare, especially when seeking care for abortion and maternal health services. Respondents were also not satisfied with the basic amenities (e.g., rest room, access to water, toilet).

We found that respondents were significantly more satisfied when care was received at home by skilled providers than other sources. In Bangladesh, community health workers who provide maternal health, child health, family planning, nutrition, and other services through door-to-door visits in the field and at home are available in all types of urban areas (e.g., 63% of city-corporation slums, 49.0% of city corporation non-slums, and 53.3% of other urban areas) [35]. In light of our study findings, increased provision of SRH services at home would likely be desirable for FSWs and might be a way to better engage them in care.

Abortion services through public, not-for-profit private (NGO) and for-profit private healthcare centers are widespread in Bangladesh. The FSWs in our study were less satisfied with abortion care received from government services compared to NGO services. A study conducted by Mannan et al. identified issues related to public abortion services, such as the distant location of health centers, financial matters (e.g., cost of transportation, unofficial fees), lack of privacy and confidentiality, cleanliness of facilities, and judgmental attitudes of service providers, and these factors resulted in lower satisfaction scores for public facilities [36]. The funding or Donor changes in NGO facilities are also a concerning issue as it affects on continuing or closing a health intervention [36].

This is the first report from Bangladesh to document the barriers to accessing care and the satisfaction levels among FSWs accessing SRH services in urban areas. However, the findings of this study may not be generalizable to FSWs in rural areas or brothel-based FSWs. The small sample sizes in the abortion (n = 77) and maternal health (n = 84) services respondent groups is another limitation. The number of FSWs who accessed care at home was also extremely small (in some cases it was only 3 FSWs). This suggests the need for larger studies in the future for better representation. It is also possible that recall or response bias influenced the findings, as all the data used was self-reported.

This main focus of this article was the barriers to accessing healthcare from a service user and provider perspective, and did not include significant reports on physical or structural barriers. Several other studies have indicated barriers such as these in SRH services for FSWs, such as lack of official documents, lack of an official recording and registration system, lack of funding or restrictive funding policies, lack of coordination among national or regional stakeholders, lack of support from government, lack of quality equipment and services, and poor accessibility to SRH information [13,29,37,38]. Key informant interviews and workshops with policymakers and program implementers should be considered to explore these barriers in future research.

Based on our findings, we have several recommendations. Firstly, an SRH-related health insurance policy could be implemented to overcome financial barriers. Developing a collaboration with health insurance companies could be considered to this effect. Secondly, a monthly training and counseling session for SRH-related service providers could encourage more compassion and friendly behavior towards FSWs. Thirdly, integration of SRH services with the DIC services should be tested so that FSWs do not feel shame to seek services, and this will also allow the adequacy of SRH services for FSWs to be confirmed. Fourthly, regular advocacy meetings with community people, religious leaders, as well as law enforcement groups should be carried out to prevent harassment by these groups. Fifthly, the existing referral system should be strengthened and sustained. Sixthly, a patient satisfaction tool should be introduced in the health facilities to monitor quality of SRH services. Finally, we recommend the urgent attention of policy makers to explore programmatic and financial sustainability of SRH services for FSWs.

## Conclusion

Our findings have indicated that FSWs experience barriers to accessing SRH. These barriers were identified by both service providers and recipients. Our findings may be particularly useful for program implementers and policy makers to take the necessary steps to overcome these barriers in the health system, in order to meet the needs of FSWs and ultimately improve their overall sexual and reproductive health.

## Supporting information

S1 File Qualitative data collection guidelines. (DOCX)

S1 Table Selected questions of questionnaire. (DOCX)
