# Supplementary material for: Barriers to sexual and reproductive healthcare services as experienced by female sex workers and service providers in Dhaka city, Bangladesh
Source: PLoS One. 2017 Jul 31;12(7):e0182249. doi: 10.1371/journal.pone.0182249 (PMC5536311; doi:10.1371/journal.pone.0182249)
Supplement: S1 File — (DOCX) [file pone.0182249.s001.docx]

**Guideline 1: In-depth interview with female sex workers**

ID of interview:

Date:

Time:

Place of interview:

DIC coverage area:

Name of interviewer:

**Background characteristics**

- What is your current age?
- Did you ever go to school? How many years had you been school?
- What is your current occupation? How long have you been engaged with current job?
- How many members do you have in your family?
- Where do you live?

**SRH practices**

***Contraceptive use***

- Have you ever heard about any contraceptive methods? What are those? Do you know where contraceptive services are available? Are you currently using any contraceptives? If yes, what type of methods are you using? Why? If you are not currently using a method, what are the reasons behind not using methods? Did you ever use any contraceptives? If yes, what type services did you use? If no, why were you not using a method ever? Did you face any problem or barrier to use a contraceptive? If yes, what were the barriers or problem that you faced (Please, probe: client side/provider side/cost/not availability/Any other)? Please, discuss details. Please, share us a story of your life that you planned to use a contraceptive but could not due to different barriers.

***Abortion***

- Have you ever heard about abortion? What do you know about abortion? Do you know where abortion services are available? Have you ever had a abortion? How many times had you have MR/Abortion? If yes, how long ago? Whom did you seek care? Why? Did you face any problem or barrier to seek care for abortion from a formal healthcare provider? If yes, what were the barriers or problem that you faced (Please, probe: client side/provider side/cost/not availability/Any other)? Please, discuss details. Please, share us a story of your life that you experienced while seeking care for abortion services.

***Maternal healthcare***

- What do you know about maternal healthcare? Do you know where maternal health services are available? If yes, please tell us about those places of maternal health care? What type of care should be received by pregnant women? Where are those care services available? Where delivery should take place? Do you know about postnatal care? If yes, please describe? Do you know about any complications that may cause maternal deaths? If yes, what are those complications? Where treatments of those complications are available? Have you ever sought care for pregnancy, delivery or postpartum after joining to sex trade? If yes, how long ago? Whom did you seek care? Where did you seek care? Please, tell us details (Probe: details care seeking on ANC/Delivery/PNC?). Did you face any problem or barrier to seek care for any maternal healthcare services from a formal healthcare provider? If yes, what were the barriers or problem that you faced (Please, probe: client side/provider side/cost/not availability/Any other)? Please, discuss details. Please, share us a story of your life that you experienced while seeking care for maternal healthcare services.

***STIs***

- Have you ever heard about sexually transmitted infections (STIs)? What do you know about STI? Do you know how can STIs be spread? Do you know how can STIs be prevented? Do you know where STI prevention or curative services are available? Have you ever had an STI)? If yes, how long ago? What type of STIs did you suffer? Whom did you seek care? Why? Did you face any problem or barrier to seek care for STI services from a formal healthcare provider? If yes, what were the barriers or problem that you faced (Please, probe: client side/provider side/cost/not availability/Any other)? Please, discuss details. Please, share us a story of your life that you experienced while seeking care for STIs services.

***Referral care***

- Have you ever been referred for any SRH care? Please, probe on each of the following issues:

- Contraceptive use (Why refer, when refer, where refer, description of any barriers faced in service utilization)

- MR/Abortion (Why refer, when refer, where refer, description of any barriers faced in service utilization)

- Maternal healthcare (Why refer, when refer, where refer, description of any barriers faced in service utilization)

- STI/AIDs (Why refer, when refer, where refer, description of any barriers faced in service utilization)

**Guideline 2: In-depth interview with DIC’s service providers**

ID of interview:

Date:

Time:

Place of interview:

DIC coverage area:

Name of interviewer:

**Background characteristics**

- What is your educational qualification?
- What is your current position in DIC?
- How long have you been engaged with current job?

**Barriers in service delivery to FSWs**

- What type of training do you have to provide DIC services? Did you receive any training after joining in your current position? If yes, please, describe details (Probe: type of training, duration, content, any more comments).
- What type of services do you provide in DIC? Please, describe details. Do you think the services you are providing to FSWs are adequate? If yes, why do you think? If no, please, describe what are the services need more to make adequate?
- What type of logistics and equipments are you required to provide your services? Do you have all required logistics and equipments? If no, please describe what type of logistics don’t you have? Please, also describe the reasons behind not having all required logistics and equipments?
- Do you need any more support from your organization to provide better services? If yes, what type of services do you need?
- Please, tell us about the whole functions of a DIC. Please probe following:
  - What type of SRH services provide by DIC to FSWs?
  - What are the modes of providing services (static/satellite clinic services)? How does it function?
  - How many human resources have in a DIC? What is their minimum qualification to get recruited? Are all required man power is available? If no, why not?
  - Do you think there are barriers to implement DIC services? If yes, what are those barriers? How to overcome those barriers?
- Do you think, FSWs are getting benefitted for DIC services? If yes or no, please describe why do you think so?
- Do you think, there are problems or constraints to provide services to FSWs? If yes, what type of problems do you think? What are your suggestions to overcome the problems or barriers?
- Do you think, there are problems or constraints to receive services by FSWs? If yes, what type of problems do you think? What are your suggestions to overcome the problems or barriers?
- Is there any referral linkage with higher level health facilities? If yes, for what type of health problems, the patients are usually refereed from a DIC? Who make the referral decision? Where does DIC refer the patients? Are there any problems or barriers to receive services from referral facilities? If yes, what are those problems? How to overcome those problems?
