# Supplementary material for: Barriers to sexual and reproductive healthcare services as experienced by female sex workers and service providers in Dhaka city, Bangladesh
Source: PLoS One. 2017 Jul 31;12(7):e0182249. doi: 10.1371/journal.pone.0182249 (PMC5536311; doi:10.1371/journal.pone.0182249)
Supplement: S1 Table — (DOCX) [file pone.0182249.s002.docx]

**Table: Selected questions from questionnaire**

| **Sl** | **Questions** | | **Response** | | | | **Code** | | **Instruction** |
| --- | --- | --- | --- | --- | --- | --- | --- | --- | --- |
|  | Contraceptive services | | | | | | | | |
| 1 | Please, tell us your current status of using contraceptive methods  (Multiple answers acceptable) | | Oral pill 1  Condom 2  IUD 3  Implant 4  Injectables 5  Female sterilization 6  Do not use anything 7  Natural methods 8  Others (Specify) …………………………….88 | | | |  | | If code=7, Go to next section (here question no 3) |
| 2 | Where do you collect contraceptive methods from?  (Multiple answers acceptable) | | Community health workers 1  Shops 2  Govt. hospitals/health centers 3  Private for profit clinic/NGOs 4  NGOs clinic/hospital 5  DIC 6  Others (Specify)……………………… ………...8 | | | |  | |  |
|  | Abortion services | | | | | | | | |
| 3 | Did you experience any abortion within last one year? | | Yes ………………………………………1  No ……………………………………….........2 | | | |  | | If no go to next  Section |
| 4 | Where did you seek care for termination of pregnancy during last time? (Multiple answers acceptable) | | a. What are the areas? | | b. What are the  sources?  (*Please, use the code below) | |  | |  |
|  |  |  | a1. In this area (in Dhaka)….....................1 | |  | |  |  |  |
|  |  |  | a2. Other area (in Dhaka)….....................2 | |  | |  |  |  |
|  |  |  | a3. Own village/native village/out of Dhaka city…………………….3 | |  | |  |  |  |
|  | *Codes for question no 4b: By skilled providers at own home=1, at skilled providers home =2, specialized hospital=3, Govt Medical College Hospital=4, District hospital=5, Maternal & Child welfare centre(MCWC)=6, Upazilla Health Complex=7,Union health and family wealth fare centre=8, Union sub-centre=9, Private Medical College Hospital =10, DIC=11, Not-for-profit NGO=12, Private for-profit clinic/hospital=13, Pharmacies=14, Doctor’s chambers=15, at own home by self/family members/neighbor/relatives=16, at own home by unskilled providers=17, at unskilled providers’ home=18, Others (Specify)=19, Don’t know=77 | | | | | | | |  |
|  | Maternal health services | | | | | | | | |
| 5 | Did you have a childbirth within last one year? | | Yes ………………………………………1  No ……………………………………….........2 | | | |  | | If no go to next  Section |
| 6 | Did you visit any health providers after being pregnant? | | Yes ………………………………………1  No ……………………………………….........2 | | | |  | | If no go to next  Section |
| 7 | Where did you receive the medical checkup from?  (Multiple answers acceptable) | | a. What are the areas? | | b. What are the  sources?  (*Please, use the code below) | |  | |  |
|  |  |  | a1. In this area (in Dhaka)….....................1 | |  | |  |  |  |
|  |  |  | a2. Other area (in Dhaka)….....................2 | |  | |  |  |  |
|  |  |  | a3. Own village/native village/out of Dhaka city…………………….3 | |  | |  |  |  |
|  | *Codes for question no 7b: By skilled providers at own home=1, at skilled providers home =2, specialized hospital=3, Govt Medical College Hospital=4, District hospital=5, Maternal & Child welfare centre(MCWC)=6, Upazilla Health Complex=7,Union health and family wealth fare centre=8, Union sub-centre=9, Private Medical College Hospital =10, DIC=11, Not-for-profit NGO=12, Private for-profit clinic/hospital=13, Pharmacies=14, Doctor’s chambers=15, at own home by self/family members/neighbor/relatives=16, at own home by unskilled providers=17, at unskilled providers’ home=18, Others (Specify)=19, Don’t know=77 | | | | | | | |  |
| 8 | Where did you give birth to (NAME)? (Single answer acceptable) | | a. What are the areas? | | b. What are the  sources?  (*Please, use the code below) | |  | |  |
|  |  |  | a1. In this area (in Dhaka)….....................1 | |  | |  |  |  |
|  |  |  | a2. Other area (in Dhaka)….....................2 | |  | |  |  |  |
|  |  |  | a3. Own village/native village/out of Dhaka city…………………….3 | |  | |  |  |  |
|  | *Codes for question no 7b: By skilled providers at own home=1, at skilled providers home =2, specialized hospital=3, Govt Medical College Hospital=4, District hospital=5, Maternal & Child welfare centre(MCWC)=6, Upazilla Health Complex=7,Union health and family wealth fare centre=8, Union sub-centre=9, Private Medical College Hospital =10, DIC=11, Not-for-profit NGO=12, Private for-profit clinic/hospital=13, Pharmacies=14, Doctor’s chambers=15, at own home by self/family members/neighbor/relatives=16, at own home by unskilled providers=17, at unskilled providers’ home=18, Others (Specify)=19, Don’t know=77 | | | | | | | |  |
|  | STI services | | | | | | | | |
| 9 | Did you experience any STDs related health problems? | | Yes ………………………………………1  No ……………………………………….........2 | | | |  | | If no go to next  Section |
| 10 | Where did you receive treatment from??  (Multiple answers acceptable) | | a. What are the areas? | | b. What are the  sources?  (*Please, use the code below) | |  | |  |
|  |  |  | a1. In this area (in Dhaka)….....................1 | |  | |  |  |  |
|  |  |  | a2. Other area (in Dhaka)….....................2 | |  | |  |  |  |
|  |  |  | a3. Own village/native village/out of Dhaka city…………………….3 | |  | |  |  |  |
|  | *Codes for question no 7b: By skilled providers at own home=1, at skilled providers home =2, specialized hospital=3, Govt Medical College Hospital=4, District hospital=5, Maternal & Child welfare centre (MCWC)=6, Upazilla Health Complex=7,Union health and family wealth fare centre=8, Union sub-centre=9, Private Medical College Hospital =10, DIC=11, Not-for-profit NGO=12, Private for-profit clinic/hospital=13, Pharmacies=14, Doctor’s chambers=15, at own home by self/family members/neighbor/relatives=16, at own home by unskilled providers=17, at unskilled providers’ home=18, Others (Specify)=19, Don’t know=77 | | | | | | | |  |
|  | Barriers to SRH care | | | | | | | | |
| 11 | Did you face any barriers to get contraceptive/ abortion/ maternal health/ STIs services from formal health sources | | Yes ………………………………………1  No ……………………………………….........2 | | | |  | | If no go to next  Section |
| 12 | If yes, what were the barriers? (Multiple answers acceptable) | | Costly/lack of money to get access to services or medication …………………………………….…1  Lack of 24 hours service availability/ seeking services during off hours…………………………………...2  Do not know where to get care…..……………….3  Distance……………………….………………...4  The unfriendly behavior of providers……………...5  Unavailability of transport….……………………6  Ashamed to seek SRH services….………………..7  Unwillingness of service providers to give services ………………………………………………….8  Hate……………………………………………..9  Others (Specify) …………………………………88  Don’t know ………………………………………77 | | | |  | |  |
|  | Satisfaction with formal healthcare | | | | | | | | |
|  | If you had sought any sexual and reproductive health care from formal health setting/providers in last one year, please, tell us your satisfaction level on quality of care as your expectation | | | | | | | | |
|  |  | a. Contraceptive use | | b. Abortion | | c. Maternal Healthcare | | d. STI | |
| 13 | Sources/places of healthcare in last one year | Public clinic/hospital..1 Public clinic/hospital (for profit) ... ... ...2  NGO clinic/hospital (not for profit).……….3  Doctor’s Chamber….4  At own home by skilled providers………...5  Skilled providers’ house…………... 6 Others……………8  Not applicable**... ..** 7  **[**if answer is ‘7’ go to next column**]** | | Public clinic/hospital.1 Public clinic/hospital (for profit) ... ... ..2  NGO clinic/hospital (not for profit).……….3  Doctor’s Chamber...4  At own home by skilled providers ...5  Skilled providers’ house……………...6 Others……………8  Not applicable**....** 7  **[**if answer is ‘7’ go to next column**]** | | Public clinic/hospital.1 Public clinic/hospital (for profit) ... ... ..2  NGO clinic/hospital (not for profit).……….3  Doctor’s Chamber...4  At own home by skilled providers ...5  Skilled providers’ house……………...6 Others……………8  Not applicable**....** 7  **[**if answer is ‘7’ go to next column**]** | | Public clinic/hospital..1 Public clinic/hospital (for profit) ... ... ...2  NGO clinic/hospital (not for profit).……….3  Doctor’s Chamber….4  At own home by skilled providers...5  Skilled providers’ house………..…... 6 Others……………8  Not applicable**... .** 7  **[**if answer is ‘7’ go to next column**]** | |
| 14 | Dignity/respect was properly maintained | Not satisfied... .... .....1 A little satisfied...... ....2 Satisfied... ... ... ... .. ....3 Moderately satisfied....4 Highly satisfied... . ... .5 Silence/no comment/could not remember... ... ..... ... 6  Not applicable ….....7 | | Not satisfied... .... ...1 A little satisfied... ....2 Satisfied... . ... ... .. ....3 Moderately satisfied.4 Highly satisfied. . ... .5 Silence/no comment/could not remember ... ..... ... 6  Not applicable ....7 | | Not satisfied... .... ...1 A little satisfied........2 Satisfied... ... ... ... .. ..3 Moderately satisfied.4 Highly satisfied... . ...5 Silence/no comment/could not remember..... ..... ... 6  Not applicable …....7 | | Not satisfied... .... ...1 A little satisfied... ....2 Satisfied... . ... ... .. ....3 Moderately satisfied.4 Highly satisfied. . ... .5 Silence/no comment/could not remember ... ..... ... 6  Not applicable ....7 | |
| 15 | Privacy was properly maintained | Not satisfied... .... .....1 A little satisfied...... ....2 Satisfied... ... ... ... .. ....3 Moderately satisfied....4 Highly satisfied... . ... .5 Silence/no comment/could not remember... ... ..... ... 6  Not applicable ….....7 | | Not satisfied... .... ...1 A little satisfied... ....2 Satisfied... . ... ... .. ....3 Moderately satisfied.4 Highly satisfied. . ... .5 Silence/no comment/could not remember ... ..... ... 6  Not applicable ....7 | | Not satisfied... .... ...1 A little satisfied........2 Satisfied... ... ... ... .. ..3 Moderately satisfied.4 Highly satisfied... . ...5 Silence/no comment/could not remember..... ..... ... 6  Not applicable …....7 | | Not satisfied... .... ...1 A little satisfied... ....2 Satisfied... . ... ... .. ....3 Moderately satisfied.4 Highly satisfied. . ... .5 Silence/no comment/could not remember ... ..... ... 6  Not applicable ....7 | |
| 16 | Autonomy (patient’s right to get information for choosing treatment options) | Not satisfied... .... .....1 A little satisfied...... ....2 Satisfied... ... ... ... .. ....3 Moderately satisfied....4 Highly satisfied... . ... .5 Silence/no comment/could not remember... ... ..... ... 6  Not applicable ….....7 | | Not satisfied... .... ...1 A little satisfied... ....2 Satisfied... . ... ... .. ....3 Moderately satisfied.4 Highly satisfied. . ... .5 Silence/no comment/could not remember ... ..... ... 6  Not applicable ....7 | | Not satisfied... .... ...1 A little satisfied........2 Satisfied... ... ... ... .. ..3 Moderately satisfied.4 Highly satisfied... . ...5 Silence/no comment/could not remember..... ..... ... 6  Not applicable …....7 | | Not satisfied... .... ...1 A little satisfied... ....2 Satisfied... . ... ... .. ....3 Moderately satisfied.4 Highly satisfied. . ... .5 Silence/no comment/could not remember ... ..... ... 6  Not applicable ....7 | |
| 17 | Confidentiality | Not satisfied... .... .....1 A little satisfied...... ....2 Satisfied... ... ... ... .. ....3 Moderately satisfied....4 Highly satisfied... . ... .5 Silence/no comment/could not remember... ... ..... ... 6  Not applicable ….....7 | | Not satisfied... .... ...1 A little satisfied... ....2 Satisfied... . ... ... .. ....3 Moderately satisfied.4 Highly satisfied. . ... .5 Silence/no comment/could not remember ... ..... ... 6  Not applicable ....7 | | Not satisfied... .... ...1 A little satisfied........2 Satisfied... ... ... ... .. ..3 Moderately satisfied.4 Highly satisfied... . ...5 Silence/no comment/could not remember..... ..... ... 6  Not applicable …....7 | | Not satisfied... .... ...1 A little satisfied... ....2 Satisfied... . ... ... .. ....3 Moderately satisfied.4 Highly satisfied. . ... .5 Silence/no comment/could not remember ... ..... ... 6  Not applicable ....7 | |
| 18 | Prompt attention | Not satisfied... .... .....1 A little satisfied...... ....2 Satisfied... ... ... ... .. ....3 Moderately satisfied....4 Highly satisfied... . ... .5 Silence/no comment/could not remember... ... ..... ... 6  Not applicable ….....7 | | Not satisfied... .... ...1 A little satisfied... ....2 Satisfied... . ... ... .. ....3 Moderately satisfied.4 Highly satisfied. . ... .5 Silence/no comment/could not remember ... ..... ... 6  Not applicable ....7 | | Not satisfied... .... ...1 A little satisfied........2 Satisfied... ... ... ... .. ..3 Moderately satisfied.4 Highly satisfied... . ...5 Silence/no comment/could not remember..... ..... ... 6  Not applicable …....7 | | Not satisfied... .... ...1 A little satisfied... ....2 Satisfied... . ... ... .. ....3 Moderately satisfied.4 Highly satisfied. . ... .5 Silence/no comment/could not remember ... ..... ... 6  Not applicable ....7 | |
| 19 | Access to social support networks during care | Not satisfied... .... .....1 A little satisfied...... ....2 Satisfied... ... ... ... .. ....3 Moderately satisfied....4 Highly satisfied... . ... .5 Silence/no comment/could not remember... ... ..... ... 6  Not applicable ….....7 | | Not satisfied... .... ...1 A little satisfied... ....2 Satisfied... . ... ... .. ....3 Moderately satisfied.4 Highly satisfied. . ... .5 Silence/no comment/could not remember ... ..... ... 6  Not applicable ....7 | | Not satisfied... .... ...1 A little satisfied........2 Satisfied... ... ... ... .. ..3 Moderately satisfied.4 Highly satisfied... . ...5 Silence/no comment/could not remember..... ..... ... 6  Not applicable …....7 | | Not satisfied... .... ...1 A little satisfied... ....2 Satisfied... . ... ... .. ....3 Moderately satisfied.4 Highly satisfied. . ... .5 Silence/no comment/could not remember ... ..... ... 6  Not applicable ....7 | |
| 20 | Basic amenities | Not satisfied... .... .....1 A little satisfied...... ....2 Satisfied... ... ... ... .. ....3 Moderately satisfied....4 Highly satisfied... . ... .5 Silence/no comment/could not remember... ... ..... ... 6  Not applicable ….....7 | | Not satisfied... .... ...1 A little satisfied... ....2 Satisfied... . ... ... .. ....3 Moderately satisfied.4 Highly satisfied. . ... .5 Silence/no comment/could not remember ... ..... ... 6  Not applicable ....7 | | Not satisfied... .... ...1 A little satisfied........2 Satisfied... ... ... ... .. ..3 Moderately satisfied.4 Highly satisfied... . ...5 Silence/no comment/could not remember..... ..... ... 6  Not applicable …....7 | | Not satisfied... .... ...1 A little satisfied... ....2 Satisfied... . ... ... .. ....3 Moderately satisfied.4 Highly satisfied. . ... .5 Silence/no comment/could not remember ... ..... ... 6  Not applicable ....7 | |
| 21 | Choice of  institution/care provider | Not satisfied... .... .....1 A little satisfied...... ....2 Satisfied... ... ... ... .. ....3 Moderately satisfied....4 Highly satisfied... . ... .5 Silence/no comment/could not remember... ... ..... ... 6  Not applicable ….....7 | | Not satisfied... .... ...1 A little satisfied... ....2 Satisfied... . ... ... .. ....3 Moderately satisfied.4 Highly satisfied. . ... .5 Silence/no comment/could not remember ... ..... ... 6  Not applicable ....7 | | Not satisfied... .... ...1 A little satisfied........2 Satisfied... ... ... ... .. ..3 Moderately satisfied.4 Highly satisfied... . ...5 Silence/no comment/could not remember..... ..... ... 6  Not applicable …....7 | | Not satisfied... .... ...1 A little satisfied... ....2 Satisfied... . ... ... .. ....3 Moderately satisfied.4 Highly satisfied. . ... .5 Silence/no comment/could not remember ... ..... ... 6  Not applicable ....7 | |
